# Mapping Evolution: Linking Adaptive Traits to Genomic Location

**DOI:** 10.1371/journal.pbio.0040275

**Published:** 2006-07-25

**Authors:** Liza Gross

Life exists in an impressive variety of forms, but the processes responsible for generating all that variation typically take far longer than the average biologist’s career. Luckily, many single-celled organisms with short generation times and prolific reproductive capacity can be easily manipulated to study the mutations associated with adapting to environmental changes.

In a new study, Ayellet Segrè, Andrew Murray, and Jun-Yi Leu describe a novel way to map evolved traits to their chromosomal location, using a genomic technique called linkage analysis. Adaptive mutations are detected based on their proximity (linkage) to neutral genetic markers (called DNA polymorphisms) observed in the ancestor. When the researchers applied their method to a parallel evolution experiment—in which four yeast strains independently adapted to alternating carbon sources—they found that each strain had acquired adaptive mutations in the same gene.

Yeast can be haploid (carry a single genome copy) and diploid (carry two copies). To generate genomic DNA for their mapping method, the researchers mated a “target” haploid yeast strain (expressing a specific trait) with a haploid “reference” strain (that lacks the trait and differs from the target strain at thousands of polymorphic sites) to create a hybrid diploid. Meiosis (cell division that turns diploid cells into haploid cells) was induced to produce a diverse assembly of recombinant offspring called segregants. From the progeny, one pool of segregants was selected that expresses the trait and a randomly collected pool served as a control.

Segrè et al. identified polymorphic sites that differed between the target and reference strains by placing DNA from each strain onto specialized microarrays called high-density oligonucleotide arrays. They chose polymorphic sites based on which oligonucleotides bound more strongly (hybridized) to target DNA than to reference DNA. (The genomic DNA is fragmented, denatured, and labeled with a fluorescent dye. Fragments that are not polymorphic relative to their complementary feature on the array light up more than polymorphic fragments.)

The method maps polymorphisms that segregate with the trait based on which oligonucleotides hybridize more strongly to DNA from trait-expressing progeny than from control progeny. After testing their method on two strains with sequenced genomes, Segrè et al. identified over 10,000 polymorphisms between their target and reference strains, called single feature polymorphisms, or SFPs—providing dense genome coverage for their linkage analysis map. (Each SFP corresponds to one oligonucleotide.) Hybridization intensities were converted into a linkage map score (LMS), which estimates the probability that the chromosomal location is linked to a selected trait.

This approach was then used to map five genes with known chromosomal locations in pools of segregants created from crossing a target strain (that can make the amino acid lysine and resist the toxicity of four drugs) and a reference strain (that lacks all five genes), and selecting for segregants that could make lysine or that could grow in a medium laced with toxic drugs. Among the highest LMS were the chromosome positions containing the five trait-related genes.

Having shown that their technique can map known genetic markers, they tested it on novel mutations underlying a trait they had evolved in laboratory yeast populations. They used four yeast populations alternately grown in media containing glucose or galactose (yeast typically grow faster on glucose) for 36 sexual cycles (about 700 cell divisions), after which a single haploid clone was selected from each population. All four strains had evolved to grow faster than their ancestors when transferred from a glucose to a galactose medium. The evolved strains, they found, overexpressed a gene encoding a positive regulator of galactose-induced transcription called *GAL3*. By genetically coupling the gene to a green fluorescent protein, they could use expression of this fusion protein to select segregants with the evolved trait and then map the trait.

In all four strains, the adaptive trait was mapped to the same region on chromosome 13, which harbors *GAL80*, encoding a repressor of galactose-induced transcription. Leu et al. sequenced the gene in the four strains and the ancestral strain and found mutations that inactivate the repressor, allowing the evolved strains to grow faster after the glucose to galactose transition. Using simulations of the mapping process, they show that their approach can work with few arrays. They also show that mapping can be done with fewer segregants, which is important for model organisms that reproduce less prolifically.

This “optimized method,” the researchers argue, can map adaptive mutations with more precision and less work (by pooling thousands of segregants) than previously described linkage approaches. And with quick, simultaneous mapping of several genes, this method could prove useful for mapping complex traits (which arise from multiple genes), an important step in understanding the genetic basis of diseases like diabetes and obesity.

## 

**Figure:**
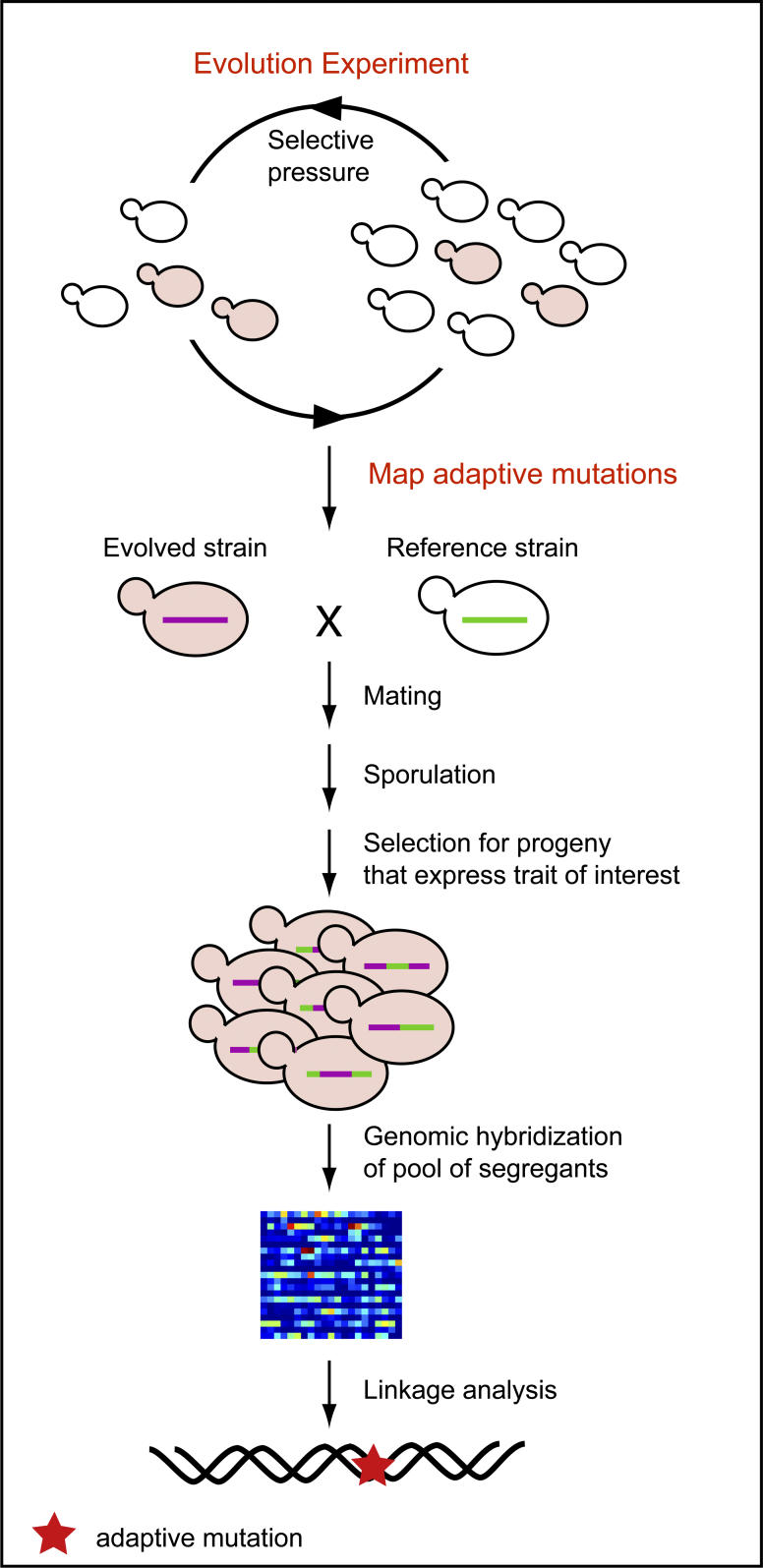
A high-resolution linkage-based mapping method can be used to study the genetic basis of experimental evolution and quantitative traits.

